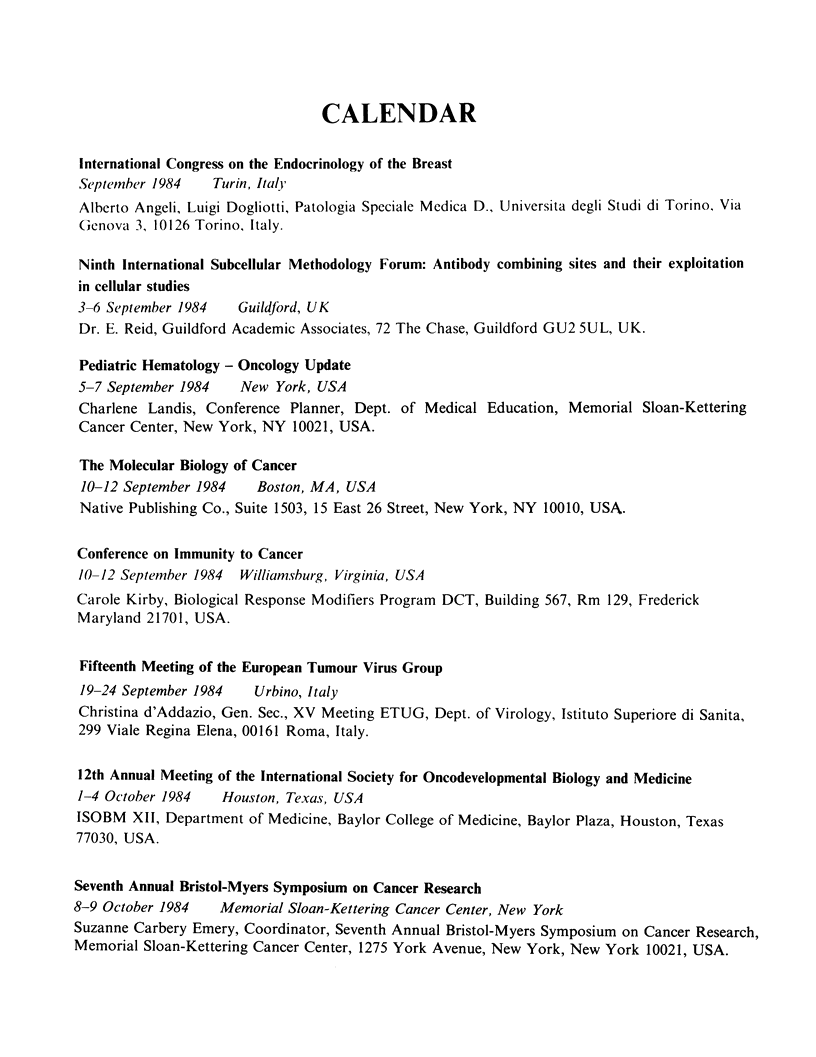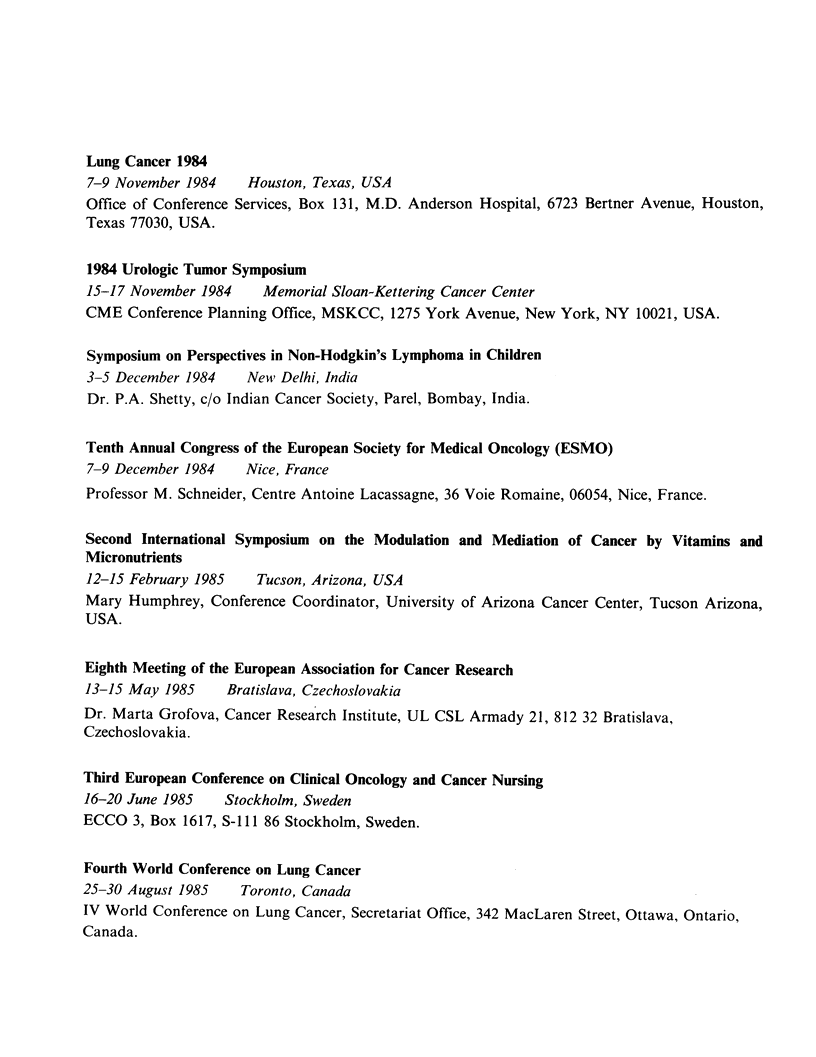# Calendar

**Published:** 1984-09

**Authors:** 


### CALENDAR

**International Congress on the Endocrinology of the Breast**
*September 1984 Turin, Italy*
Alberto Angeli, Luigi Dogliotti, Patologia Speciale Medica D., Universita degli Studi di Torino, Via Genova 3, 10126 Torino, Italy.

**Ninth International Subcellular Methodology Forum: Antibody combining sites and their exploitation in cellular studies**
*3–6 September 1984 Guildford, UK*
Dr. E. Reid, Guildford Academic Associates, 72 The Chase, Guildford GU2 5UL, UK.

**Pediatric Hematology – Oncology Update**
*5–7 September 1984 New York, USA*
Charlene Landis, Conference Planner, Dept. of Medical Education, Memorial Sloan-Kettering Cancer Center, New York, NY 10021, USA.

**The Molecular Biology of Cancer**
*10–12 September 1984 Boston, MA, USA*
Native Publishing Co., Suite 1503, 15 East 26 Street, New York, NY 10010, USA.

**Conference on Immunity to Cancer**
*10–12 September 1984 Williamsburg, Virginia, USA*
Carole Kirby, Biological Response Modifiers Program DCT, Building 567, Rm 129, Frederick Maryland 21701, USA.

**Fifteenth Meeting of the European Tumour Virus Group**
*19–24 September 1984 Urbino, Italy*
Christina d'Addazio, Gen. Sec., XV Meeting ETUG, Dept. of Virology, Istituto Superiore di Sanita, 299 Viale Regina Elena, 00161 Roma, Italy.

**12th Annual Meeting of the International Society for Oncodevelopmental Biology and Medicine**
*1–4 October 1984 Houston, Texas, USA*
ISOBM XII, Department of Medicine, Baylor College of Medicine, Baylor Plaza, Houston, Texas 77030, USA.

**Seventh Annual Bristol-Myers Symposium on Cancer Research**
*8–9 October 1984 Memorial Sloan-Kettering Cancer Center, New York*
Suzanne Carbery Emery, Coordinator, Seventh Annual Bristol-Myers Symposium on Cancer Research, Memorial Sloan-Kettering Cancer Center, 1275 York Avenue, New York, New York 10021, USA.

**Lung Cancer 1984**

*7–9 November 1984* *Houston, Texas, USA*

Office of Conference Services, Box 131, M.D. Anderson Hospital, 6723 Bertner Avenue, Houston, Texas 77030, USA.

**1984 Urologic Tumor Symposium**

*15–17 November 1984* *Memorial Sloan-Kettering Cancer Center*

CME Conference Planning Office, MSKCC, 1275 York Avenue, New York, NY 10021, USA.

**Symposium on Perspectives in Non-Hodgkin's Lymphoma in Children**

*3–5 December 1984* *New Delhi, India*

Dr. P.A. Shetty, c/o Indian Cancer Society, Parel, Bombay, India.

**Tenth Annual Congress of the European Society for Medical Oncology (ESMO)**

*7–9 December 1984* *Nice, France*

Professor M. Schneider, Centre Antoine Lacassagne, 36 Voie Romaine, 06054, Nice, France.

**Second International Symposium on the Modulation and Mediation of Cancer by Vitamins and Micronutrients**

*12–15 February 1985* *Tucson, Arizona, USA*

Mary Humphrey, Conference Coordinator, University of Arizona Cancer Center, Tucson Arizona, USA.

**Eighth Meeting of the European Association for Cancer Research**

*13–15 May 1985* *Bratislava, Czechoslovakia*

Dr. Marta Grofova, Cancer Research Institute, UL CSL Armady 21, 812 32 Bratislava, Czechoslovakia.

**Third European Conference on Clinical Oncology and Cancer Nursing**

*16–20 June 1985* *Stockholm, Sweden*

ECCO 3, Box 1617, S-111 86 Stockholm, Sweden.

**Fourth World Conference on Lung Cancer**

*25–30 August 1985* *Toronto, Canada*

IV World Conference on Lung Cancer, Secretariat Office, 342 MacLaren Street, Ottawa, Ontario, Canada.